# A New Look at Vaccine Strategies Against PPRV Focused on Adenoviral Candidates

**DOI:** 10.3389/fvets.2021.729879

**Published:** 2021-09-08

**Authors:** José M. Rojas, Noemí Sevilla, Verónica Martín

**Affiliations:** Centro de Investigación en Sanidad Animal (CISA-INIA-CSIC), Instituto Nacional de Investigación y Tecnología Agraria y Alimentaria, Consejo Superior de Investigaciones Científicas, Madrid, Spain

**Keywords:** PPRV, vaccines, adenovirus, viral vector, immune response

## Abstract

Peste des petits ruminants virus (PPRV) is a virus that mainly infects goats and sheep causing significant economic loss in Africa and Asia, but also posing a serious threat to Europe, as recent outbreaks in Georgia (2016) and Bulgaria (2018) have been reported. In order to carry out the eradication of PPRV, an objective set for 2030 by the Office International des Epizooties (OIE) and the Food and Agriculture Organization of the United Nations (FAO), close collaboration between governments, pharmaceutical companies, farmers and researchers, among others, is needed. Today, more than ever, as seen in the response to the SARS-CoV2 pandemic that we are currently experiencing, these goals are feasible. We summarize in this review the current vaccination approaches against PPRV in the field, discussing their advantages and shortfalls, as well as the development and generation of new vaccination strategies, focusing on the potential use of adenovirus as vaccine platform against PPRV and more broadly against other ruminant pathogens.

## Introduction

Due to the risk of viral escape mutants from antiviral treatments as well as the excessive use of antibiotics that causes the appearance of bacterial resistances, vaccination continues to be one of the best measures to prevent infectious diseases. Since Jenner's time, vaccines have come a long way thanks to the development of knowledge and technology in molecular biology and immunology.

The relevance and impact of a pandemic due to a human pathogen is not the same as that of diseases in ruminants. But, precisely because we are living in a globalized world, in which pathogens jump more and more frequently from animals to humans, animal health should be a priority. This is framed within the One Health concept, in which animal health and human health are interdependent, and global strategies to prevent and control pathogens must be implemented.

Among the diseases of relevance in animal health, peste des petits ruminants (PPR) stands out. It is caused by the peste des petits ruminants virus (PPRV) and affects mainly small domestic ruminants (sheep and goats) as well as camels, with serious economic loss especially in many countries of Africa and Asia (1, 2). Wild ruminants, such as gazelles, deer, roe deer, antelope can also be affected (3–12), which consequently poses a further risk for the control and surveillance in vaccination programs.

PPRV belongs to the genus *Morbillivirus* among which are included the important human pathogen measles virus (MV), as well as veterinary pathogens such as canine distemper virus (CDV), feline morbillivirus, dolphin and porpoise morbillivirus (DMV, PMV), phocine distemper virus (PDV), morbilli-like bat or rodents virus, and the eradicated rinderpest virus (RPV)

(13–17). Rinderpest virus (RPV) vaccination has shielded PPRV from visibility for years, as it provided partial protection against PPRV, but once RPV was eradicated and vaccination programs stopped in 2011 PPRV emergence became clearly evident. In 2014, the World Organization for Animal Health (OIE) considered PPRV the second animal pathogen candidate to be eradicated, establishing an eradication program aimed at 2030. To achieve this, synergies must be produced between governments, researchers, companies and farmers (18).

The vaccines currently used in the field against PPRV are live attenuated vaccines. Despite the fact that these vaccines generate protection, they present drawbacks that need addressing. Importantly, the eradication program would be greatly helped with the development of vaccines that allow differentiation of infected from vaccinated animals (so-called DIVA vaccines), which would facilitate the control and surveillance programs in the vaccinated areas. Likewise, vaccines that are independent of a cold chain for their preservation, one of the main drawbacks of live attenuated vaccines, would be advantageous since in most countries where PPRV is endemic, the maintenance of cold storage and transport facilities can be problematic and could lead to vaccine administration in poor immunization conditions. These are achievable goals as we have seen in these times of the Covid-19 pandemic, where the reaction capacity of pharmaceutical companies and the scientific community has successfully developed several vaccines, based on different technologies.

**Figure 1:**
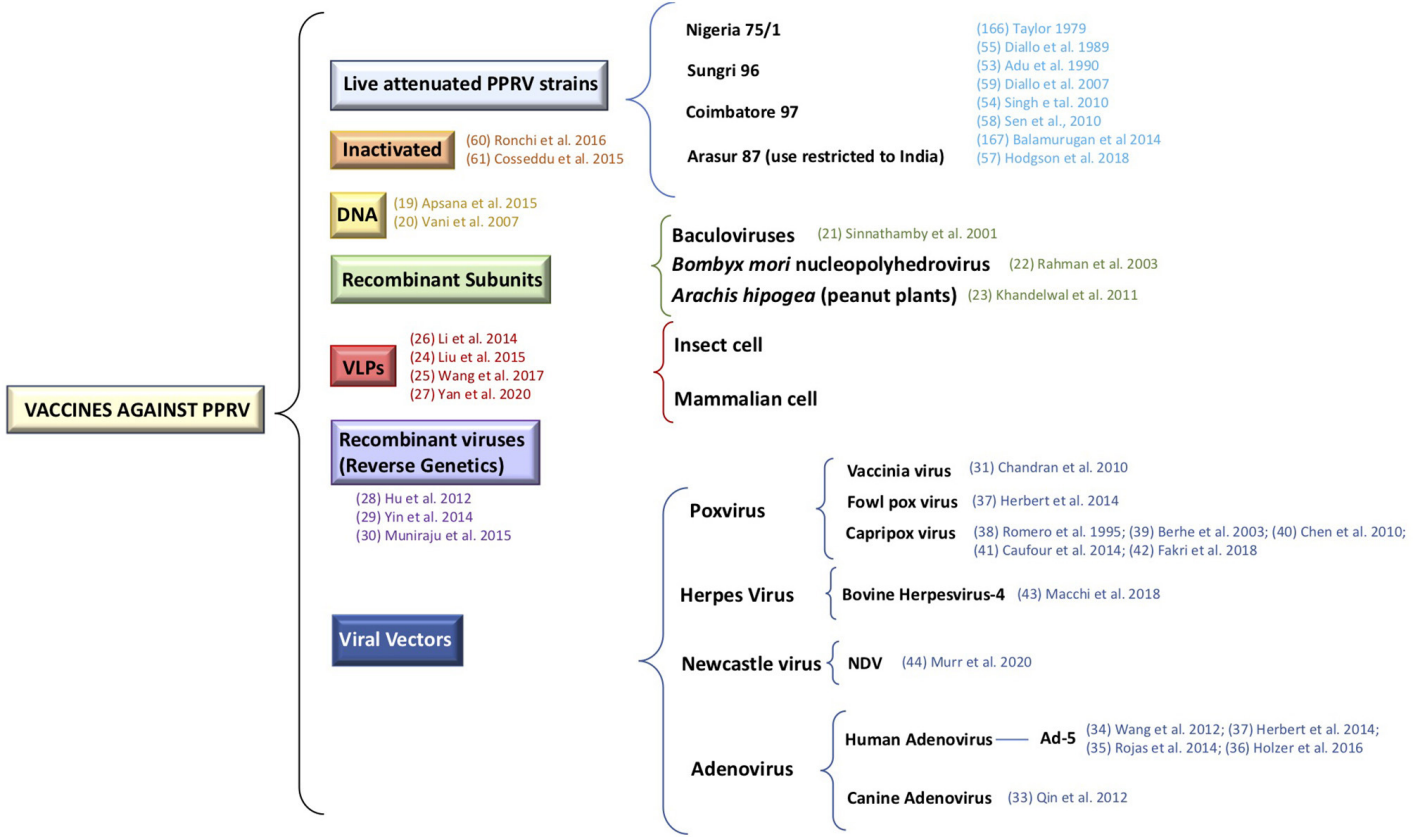
Representation of the different vaccines strategies against PPRV.

Currently, several laboratories are working on different approaches, aimed at overcoming the aforementioned weak points of current vaccines, by developing a new generation of vaccines against PPRV. These alternative approaches include inactivated vaccines, DNA vaccines (19, 20), recombinant subunit vaccines (21–23), virus-like-particles (VLPs) vaccines (24–27), reverse-genetic vaccines (28–30), and vectored vaccines (31–44) (Figure 1).

We summarize in this review the current vaccines available against PPRV in the field as well as the development and generation of new vaccination strategies, focusing on the potential use of adenovirus as vaccine platform against this pathogen.

## PPRV Characteristics

**Figure 2:**
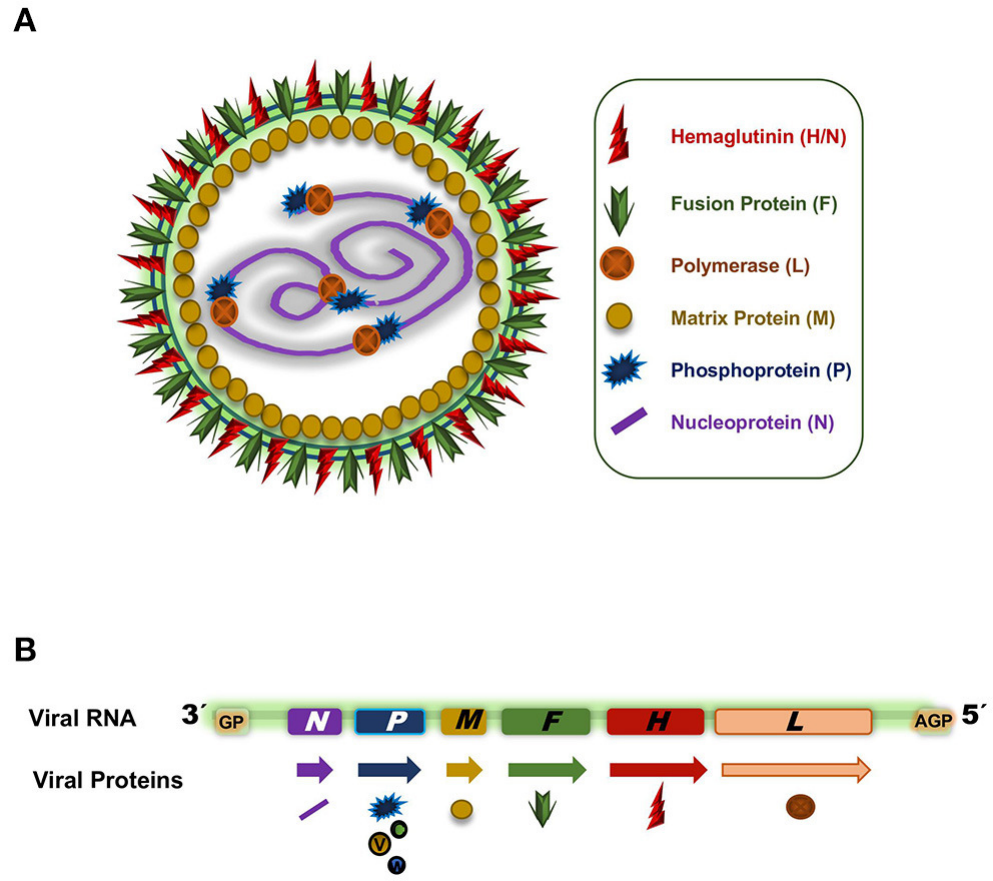
**(A)** PPRV capsid representation. **(B)** PPRV RNA genome. Arrows indicate the different transcripts and the symbols indicate the proteins codified from the different genes.

PPRV is an RNA virus classified into the order *Mononegavirales, Paramyxoviridae* family and genus *Morbillivirus*. It is a polymorphic enveloped negative single strand RNA virus with two external glycoproteins decorating the envelope, the fusion protein (F) and the hemagglutinin (H) (45). The viral particle size ranges from 400 to 500 nm (45). The non-segmented RNA molecule is packaged in a ribonucleoprotein complex (RNP) inside the envelope with the nucleoprotein (N), the phosphoprotein (P), and the RNA polymerase (L) (Figure 2A). The RNP adopts a helical structure and a single particle can incorporate more than one RNP, thus making PPRV polyploid (46). Associated with the inner surface of the plasma membrane and the cytoplasmic tails of F and H glycoproteins is the matrix protein (M). In addition to the six structural proteins mentioned (F, H, N, P, L, M), the single-stranded RNA molecule encodes two non-structural proteins, termed C and V, as well as a putative protein W (Figure 2B).

PPRV is genetically grouped into four distinct lineages (I, II, III, and IV) on the basis of partial sequence analysis of fusion protein (F) gene or nucleoprotein (N), but only one serotype exists (47–49). Although there is cross-protection between lineages, it is interesting to classify them for control and epidemiological studies, thus allowing source tracing of outbreaks. All PPRV strains present in Asia belong to the genetic lineage IV (50), which was reported for the first time in Africa, during the Morocco outbreak in 2008 (51). All four lineages are prevalent in Africa. Lineages I and II have been reported in West Africa, whereas lineage III has been found in eastern Africa, Arabian Peninsula and southern India (48).

## Current Vaccines Against PPRV in The Field

### Live Attenuated Vaccines

PPRV strains obtained from different circulating lineages and attenuated by several passages in tissue culture have been traditionally used in Africa, the Middle East and many countries in Asia as vaccines against PPR. Gilbert and Monnier were the first to adapt the virus to cell cultures, performing serial passages (12) in sheep embryo kidney epithelial cells and using for their first passage blood extracts from animals infected with PPRV (52). The Nigeria 75/1 virus of lineage II, together with the Sungri 96 of lineage IV, obtained after 63 or 75 successive passages in the Vero cell line, respectively, are the most frequently used commercial attenuated vaccines for PPRV in endemic countries (53–56). The two other live attenuated vaccines currently available are Coimbatore 97 and Arasur 87, which are restricted to India.

For a long time, the generation of a humoral immune response by a vaccine candidate was considered sufficient and the most important correlate of vaccination with protection. For some years now, the generation of a cellular response to PPRV has been considered almost as essential as the humoral one. Curiously, goats vaccinated with Nigeria 75/1 develop a greater antibody response than those vaccinated with Sungri 96. Conversely, Sungri 96 vaccination was more efficient at activating a cellular response with increase IFN-γ production and lymphocyte proliferation against PPRV and a higher number of CD4+ T lymphocytes (57). In spite of these differences in cellular and humoral immune responses, both vaccine strains are capable of protecting equally well-against PPRV challenge from the 4 different lineages (57).

These live attenuated vaccines generate a long-lasting immunity to PPRV that lasts for at least 3 years post-vaccination (58, 59). Thus, protection against the circulating lineage and cross-protection between lineages is obtained with these vaccines. The protection is based on potent humoral and cellular immune responses. However, these vaccines do not allow to differentiate vaccinated from infected animals and thus they are not DIVA vaccines. Moreover, although it is a rare event, virus reversion from attenuated to virulent can occur, which could potentially cause an outbreak. Therefore, from the point of view of epidemiological control and surveillance, live attenuated vaccines are not the most desirable despite their effectiveness, particularly for epizootic outbreaks. Additionally, the effectivity of these vaccines depends on the cold-chain preservation. Therefore, great efforts have placed into developing alternative to live attenuated vaccines to fulfill the need for DIVA and thermotolerant vaccines for PPRV.

## Potential Vaccines Against PPRV

### Inactivated Vaccines

Inactivated virus vaccines offer the advantage of increased safety that comes nonetheless at the cost of loss of immunogenicity. As a result, addition of adjuvant is usually required for these inactivated vaccine formulations. In non-endemic regions inactivated vaccine is often preferred as it eliminates the risk of reversion and disease spreading of the live attenuated vaccines. An inactivated PPRV vaccine has been described based on the lineage IV Moroccan PPRV strain M/08 that was isolated from a deceased goat during the 2008 PPRV outbreak on Morocco (60). Inoculation of this binary ethyleneimine inactivated virus was safe in rats and goats and induced humoral responses (60). A transient seroconversion at day 9 until day 30 post first immunization was induced in goats, that after a booster immunization (day 36) was converted into a robust and persistent seroconversion with PPRV neutralizing antibody responses until, at least, day 110 post-booster (60, 61). This inactivated PPRV vaccine protects the natural host against homologous virus challenge (61). As often is the case with inactivated virus vaccines, adjuvant addition was necessary to boost the immunogenicity of the formulation (60) (Supplementary Table 1). There are yet no data on the cellular immune response induced by this inactivated vaccine. Nevertheless, this approach remains attractive and is more readily acceptable for veterinary authorities particularly in non-endemic regions.

### DNA Vaccines

DNA vaccines are often thought of as an alternative to conventional vaccines due to their relative ease of production and their stability at room temperature. A PPRV DNA vaccine candidate based on a *Semliki Forest virus* replicon expressing the PPRV-F or -H genes has been shown to induce cellular and humoral responses in a murine model (62, 63). However, the immunogenicity of these constructs has however yet to be tested in the natural host. DNA vaccination using plasmids that express the anti-idiotypic determinants of PPRV-H protein as an antigen mimic has also been employed to induce immunity to PPRV (19, 64). This DNA vaccination regime elicited cellular and neutralizing antibody responses in sheep although protection has to be addressed (19) (see Supplementary Table 1).

### Recombinant Subunit Vaccines

Another approach to vaccination is the use of recombinant systems to express an antigenic viral protein that will be formulated for vaccination. Insect baculoviruses have been used as a display system for PPRV immunogenic proteins. Inoculation of recombinant baculovirus expressing PPRV-H in the envelope induces cellular immunity and PPRV neutralizing antibodies in goats (65). A recombinant *Bombyx mori* nucleopolyhedrovirus expressing the PPRV-F and RPV-H proteins was capable of eliciting neutralizing antibodies to both viruses when inoculated in mice (22). Other recombinant protein expression systems have also been studied. For instance, an alternative immunization strategy using recombinant PPRV-H protein expressed in transgenic peanut plants and fed orally to sheep produced anti-PPRV neutralizing antibodies and specific T cell responses to PPRV-H protein in sheep (23). Overall, although these PPRV antigen delivery systems can elicit immunity to the virus, their potency as vaccine has yet to be established (Summarized in Supplementary Table 1).

### Virus-Like-Particles (VLPs) Vaccines

Another attractive strategy for vaccine design consists in immunizations with virus-like particles, i.e., providing the capsid antigens of the virus to the immune system for recognition without the viral genetic material. These vaccination systems are deemed extremely safe, as viral replication cannot occur in the absence of genetic material, but they often require the addition of an adjuvant to boost immunogenicity. PPRV VLP production has been described using different expression systems such as insect cells or mammalian Vero cells (25, 26). It appears that the matrix protein M is critical to the formation of PPRV VLPs (25), whereas inclusion of major neutralizing antibody determinants like the PPRV-H protein likely promote the immunogenicity of these VLPs. Some of these VLP constructs have proved to be immunogenic in mice eliciting humoral immunity (24). Immunogenicity in goats of PPRV VLPs has also been confirmed (26, 66). In these studies VLP vaccination elicited neutralizing antibodies and cellular immune responses even in the absence of adjuvant in some reports (26). Recently, a PPRV VLP based on the virulent lineage IV Tibet/30 isolate was shown to induce stronger immune responses in goats and sheep than VLPs produced from the vaccine strain Nigeria 75/1 (27) indicating that this strategy could be applied to new virulent isolates. Overall, PPRV VLPs could be a promising vaccine candidate in spite of the likely necessity to supplement the formulation with adjuvant to boost immunogenicity. These VLPs have also the potential to be DIVA vaccines as they only express some of the viral gene products. Further studies are nonetheless required to demonstrate their protective efficacy against virulent PPRV challenge. Comparative VLPs vaccination details are summarized in Supplementary Table 1.

### Reverse-Genetics-PPRV

Reverse genetic has been an important advance in virology. Through genetic engineering, this technique makes it possible to entirely obtain a recombinant virus from full-length complementary DNA copies (cDNA) of the viral RNA genome. Reverse genetic systems have provided the vaccine field with a powerful technology to generate, with a more rational approach, different types of vaccines that can be positively or negatively marked. Despite PPRV, like all morbillivirus, being an easy candidate and target for this technique, and having a minigenome described since 2007 (67), it was not possible to recover a recombinant PPRV based on reverse genetics until 2012 (28). Hu et al. generated a stable recombinant GFP-expressing PPRV virus that allowed for the development of high throughput fluorescence-based seroneutralization tests (28). Moreover, this genetic mark introduced into the vaccine makes it possible to differentiate between infected and vaccinated animals. Some years later, the PPRV vaccine strain Nigeria 75/1 was also modified by reverse genetics to eliminate a B cell epitope from PPRV-H protein that is recognized by a monoclonal antibody used in anti-H ELISA to attempt to produce a DIVA live-attenuated vaccine (30). The genetically modified recombinant vaccine strain showed similar vaccination potency as the PPRV Nigeria 75/1 vaccine both in terms of induction of humoral immunity and in providing protection against virulent PPRV challenge in goats. However, the mutations introduced in PPRV-H to avoid antibody recognition were not sufficient to differentiate infected from vaccinated goats using an anti-H ELISA kit (30). Reverse genetics can also be used to generate multivalent vaccines by expressing antigens from other diseases. Using the PPRV Nigeria 75/1 vaccine strain backbone, a recombinant PPRV vaccine expressing the VP1 structural protein from foot and mouth disease virus (FMDV) has been developed (29). This recombinant vaccine was capable of inducing neutralizing antibodies against both PPRV and FMDV. Moreover, it was able to provide partial protection against FMDV infection (29), indicating that this molecular strategy has the potential to develop bivalent vaccines for ruminant diseases. Using reverse genetics, live attenuated PPRV vaccines could also be modified to remove additional virulence factors to further improve their safety. Reverse genetics recombinant PPRV vaccines share same advantages and disadvantages with live attenuated vaccines, i.e., they are likely to provide long-term PPRV immunity but still present the risk of reversion.

### Vector Vaccines for PPRV

One of the most promising approaches to generate DIVA vaccines for PPRV is the use of recombinant viral vectors that express PPRV immunogenic proteins. Research aimed at generating vaccines based on different viral vectors is abundant. The H and F glycoproteins are the principal targets of neutralizing antibodies of the humoral immune response. These two glycoproteins have been considered the best to include in vaccine candidates and their genes have been cloned and expressed in several recombinant, replication-defective viral vectors: (i) Poxviruses (39, 40), (ii), Bovine-Herpes viruses (43), (iii) Newcastle disease virus (44), and (iv) Adenoviruses (33, 34, 36, 37, 68).

Another strategy is to generate multivalent vaccines, i.e., a vaccine that can protect against several diseases. Some of the viral vectors that have been chosen as carriers of the PPRV H and /or F genes come from viruses that are also causative of disease in animals. For this reason, bivalent vaccines are desirable so that they generate an immune response in sheep or goats capable of protecting against PPRV and at the same time against the disease caused by the virus onto which the vaccine vector is based.

#### Poxvirus Vectors

Poxviral vectors can harbor large DNA inserts; do not integrate into the host genome due to their cytoplasmic replication, and importantly, induce cellular and humoral immunity to the inserted transgene. These characteristics have boosted the interest for these vectors in vaccinology (69, 70). Several vectors derived from this family have been used for PPRV vaccination.

##### Vaccinia Vectors

Vaccinia vectors were the first recombinant vectors to show efficacy in vaccination against PPRV. Jones et al. showed in 1993 that a vaccinia virus vector based on the Wyeth strain and made to express the proteins F and H from RPV could protect goats against virulent PPRV challenge (71). Interestingly this protection occurred in spite of the vaccination failing to trigger detectable levels of neutralizing antibodies to PPRV. In a different study, goats were protected against a virulent Indian PPRV strain challenge when vaccinated with recombinant attenuated Modified Vaccinia Ankara (MVA) viruses expressing F or H PPRV genes (31). In this case vaccination correlated with neutralizing antibody induction. Cellular immunity induced by these MVA vaccines was not assessed in the study.

##### Fowl Pox Vectors

Fowl pox vectors expressing PPRV F or H protein have also been generated (37). Avian poxviruses present the safety advantage of being unable to replicate in mammalian cells, but are still able to infect and express the transgene of interest. Vaccination with these recombinant vectors triggered cellular immunity to PPRV-F or -H, but failed to induce significant neutralizing antibody levels (37).

##### Capripox Vectors

Capripoxvirus is the causal agents of the contagious diseases goatpox and sheeppox, the distribution of which often overlaps with regions where PPRV is endemic. Using attenuated capripox virus vaccine strains as the recombinant vector basis, bivalent vaccines that elicit protection against goatpox/sheeppox and PPRV have been developed. Introduction of H or F genes from PPRV conferred protective immunity against PPRV in goats (39, 40, 42). Indeed, even the expression of H or F genes from RPV conferred protection against PPRV infection in goats using this system (38). Some studies, nonetheless, highlighted a possible shortfall of this bivalent strategy: existing immunity to the capripoxvirus vector could limit the immunity induced to the expressed PPRV gene. Caufour et al., found that animals with pre-existing immunity to capripoxvirus developed only partial protection against PPRV (41). This was however not observed in another study indicating that this drawback can probably be overcome with a booster vaccination (42). Overall, poxvirus-based vaccine strategies appear promising. These vectors have often been described to work better in heterologous prime-boost strategies (with other recombinant vectors or with DNA for instance). These approaches have yet to be evaluated for PPRV.

#### Bovine-Herpes Viruses

Bovine Herpesvirus-4 (BoHV-4) can replicate in a broad range of host species, but only produces subclinical infections in cattle (72). Recombinant BoHV-4 vectors can also induce potent host immune responses (73), while generating low levels of neutralizing antibodies against the vector (74). A recombinant BoHV-4 expressing the PPRV H protein from Nigeria 75/1 strain induced a potent humoral and cellular immune response in mice (43), as well as in sheep, conferring protection against an heterologous virulent PPRV challenge (75). This recombinant vaccine could represent an attractive platform for PPRV vaccination.

#### Newcastle Disease Virus (NDV)

Recently a recombinant Newcastle disease virus expressing PPRV H protein was shown to induce protective immunity against virulent PPRV challenge in goats (44). NDV is a Paramyxovirus that produces diseases in poultry, but attenuated strains are used as vaccine. These viruses have a broad spectrum of infectivity but their replication is limited in mammalian host cells, thus, raising their safety profile when used as a recombinant vector in mammals. Dual injection of NDV expressing PPRV-H induced a similar degree of protection against challenge as vaccination with the conventional Nigeria 75/1 vaccine (44), indicating that NDV-based vector could be useful for PPRV control.

#### Adenovirus Vectors: the Vector of Choice for a PPRV DIVA Vaccine?

Vaccine developments against the SARS-CoV2 pandemic have put adenoviral vectors at the forefront for vaccine design. This is clearly exemplified by the approval by health authorities of several vaccine formulations against Covid-19 based on adenoviral vectors (76). Vaccination with adenoviral vectors expressing the immunogenic proteins F or H from PPRV have also shown promising results in protection studies in the natural hosts of PPR (35–37). In this section of the review we will describe in more details adenovirus vectors and discuss their advantages and limitations for vaccine development focusing on their veterinary use and more precisely on PPRV.

## Types of Adenovirus Vectors

**Figure 3:**
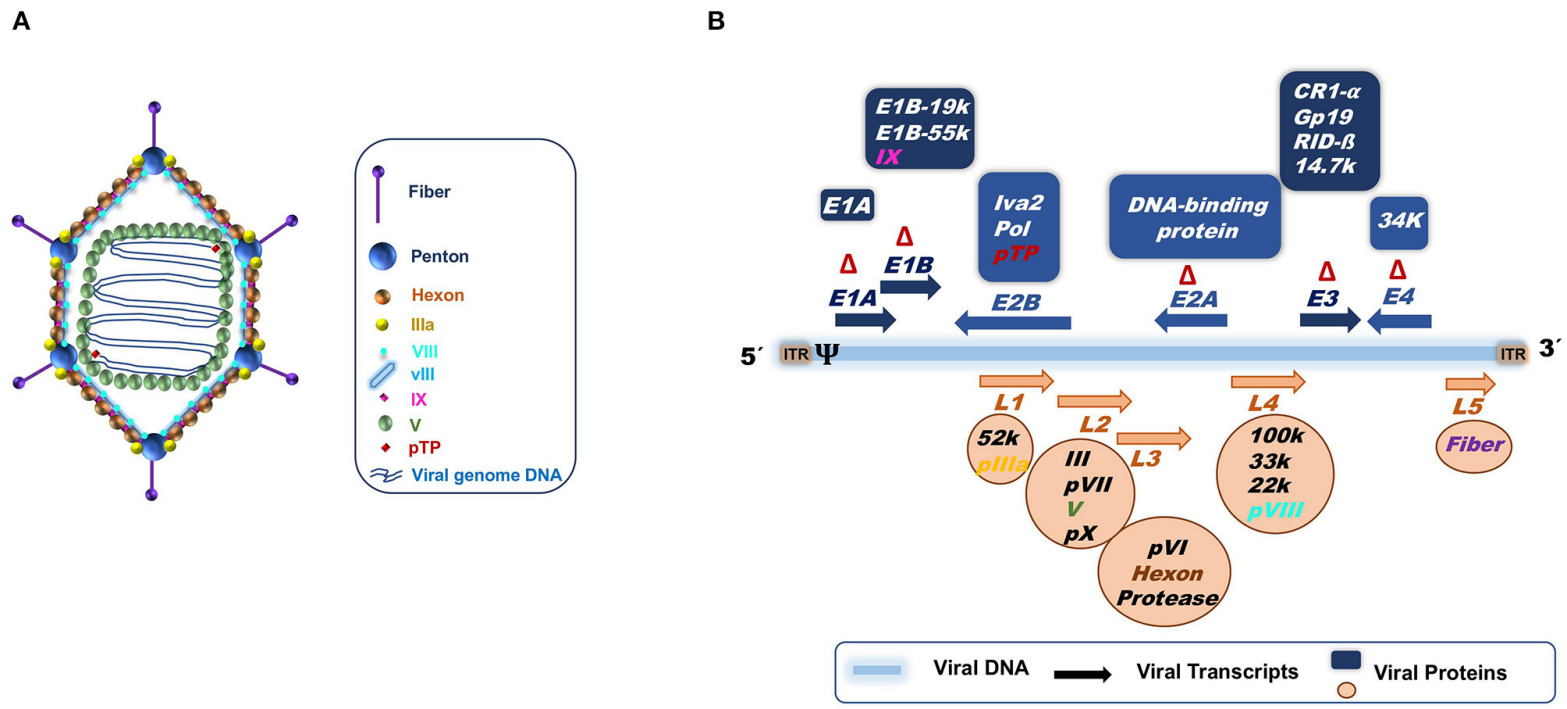
**(A)** Adenoviral capsid. **(B)** Adenoviral genome structure based on Ad5 knowledge. The 36 Kb double stranded DNA is represented by a blue line. Arrows indicate the transcription units. Early transcript units (E1 to E4) are indicated in blue above the DNA, while late transcription units (L1 to L5) are represented in orange below the DNA. The orientation of the arrows signifies the gene transcription direction, right to left (→) or left to right (←). The proteins codified for the different transcripts are specified in the blue boxes for early genes and in orange circles for late genes. ψ, is the packaging signal; ITR, Internal terminal repeats; Δ, Deletions for the constructions of the different adenoviral vectors; first (ΔE1 and ΔE3) and second generation (ΔE1 and ΔE3 plus ΔE2 and ΔE4). In the case of “gutless” adenoviral vectors, the ITRs and the packaging signal (ψ) are the only adenoviral genome parts that remain.

Adenoviruses are 35–40 kb dsDNA genome, non-enveloped viruses (Figure 3). Infection with adenoviruses usually provokes common flu-like disease in humans and animals, during which the neutralizing antibody response controls the infection. They have demonstrated to be excellent candidates as vaccine delivery vehicles (77). Particles can be engineered as replicating or as defective in replication. They are safe, genetically stable and manufacturable in high amounts (78, 79).

The type of adenoviral vectors that have been engineered can be summarized into three categories depending on the amount of adenovirus genome deleted to allow insertion of transgenes (Figure 3B). The first generation of adenoviral vectors, in which the area of the adenoviral genome corresponding to E1 and E3 regions are deleted (ΔE1)(ΔE3), renders them replication-defective, thus able to infect host cells but unable to replicate (ΔE1) (80, 81). Furthermore, since the E3 genes have been related to adenovirus immune evasion mechanisms, the deletion of the E3 region improves the immune response to the adenovirus (82, 83). The second generation incorporates additional deletions or inactivated zones in the adenoviral genome, corresponding to E2 and E4 regions that code for proteins involved in viral replication in target cells (84–86), increasing vector safety by avoiding the generation of replication competent adenovirus by recombination (87). This comes also at the cost of diminishing vector immunogenicity (83, 88). Finally, the third adenoviral vector generation eliminates the entire adenoviral genome except the ITRs and the packaging signal. These are called “gutless” or helper dependent vectors (89–92). The “gutless” adenoviruses allow the immune response to be directed mainly against the transgene instead of the vector, but also causes a decrease in the adjuvant effect provided by the adenoviral vector itself. The second and third adenoviral generation vectors are more difficult to produce in high amounts.

The increase in deleted adenoviral genome regions increases the acceptance size of heterologous genes, from 4.5 kb for the first generation, to 10 kb for the second generation, and finally to 36 kb for the third generation. This provides the adenoviral vectors with the features of vehicle for delivery of antigens by expressing the transgenes in the target cells. Transgene expression is transitory and lasts for 2–3 weeks, as proved in different animal and human models. This generates a strong immune response consisting of CD4+ and CD8+ T cell directed against the transgene and also against adenoviral antigens (except in “gutless” vectors) (93–100). Replication-defective adenoviruses need to be obtained through transfection of HEK293 or Per.C cells which provide in *trans* the E1 and/or E2 and E4 functions (101–105). The “gutless” vector additionally requires the presence of a helper adenovirus, rendering the production system more complex. For the second and third generation vectors, the complementation is not as efficient as desired in the producer cell lines (85, 106), reducing the yields obtained. Although they have benefits, such as less immunogenicity and less cellular toxicity (107–111). Recombinant first generation adenoviruses are thermotolerant and not difficult to produce in large quantities and thus, they can be easily transported without losing their immunogenicity to endemic areas of PPRV, which coincide with hot climate areas in the world (Africa and Asia).

Adenoviruses can be engineered to be replication incompetent or to remain competent in replication, expressing in both cases a foreign gene. Both of them present advantages and disadvantages. Replication incompetent adenoviruses are elected primarily as vaccine candidates because the majority of the immune response they trigger is targeted to the transgene they expressed. The adenoviral protein expression is limited as it is overtaken by the transgene expression (112). They are also safer, as they cannot replicate and thus spread to a different host. In contrast, the competent replication adenovirus vectors enhance the immune response (113, 114) against both the vector and the transgene, which could limit the vaccine efficacy due to vector neutralization. They can also cause serious issues in immunosuppressed individuals due to possible vector-derived pathologies, as well as escape of potentially virulent revertant viruses. For these reasons, it is more difficult to obtain approval by competent authorities to bring the replication-competent vectors to the market. However, in the veterinary field these replication competent vectors could have some applications. For instance, a competent replicative adenoviral vector expressing the rabies virus glycoprotein has been successfully delivered to wildlife through baiting for rabies control campaigns in Canada (115). This vaccine has proved to be safe in a number of species with minimal risk of horizontal transmission (116).

## Adenoviral Vector: Immunity in Response to Viral Vectors

**Figure 4:**
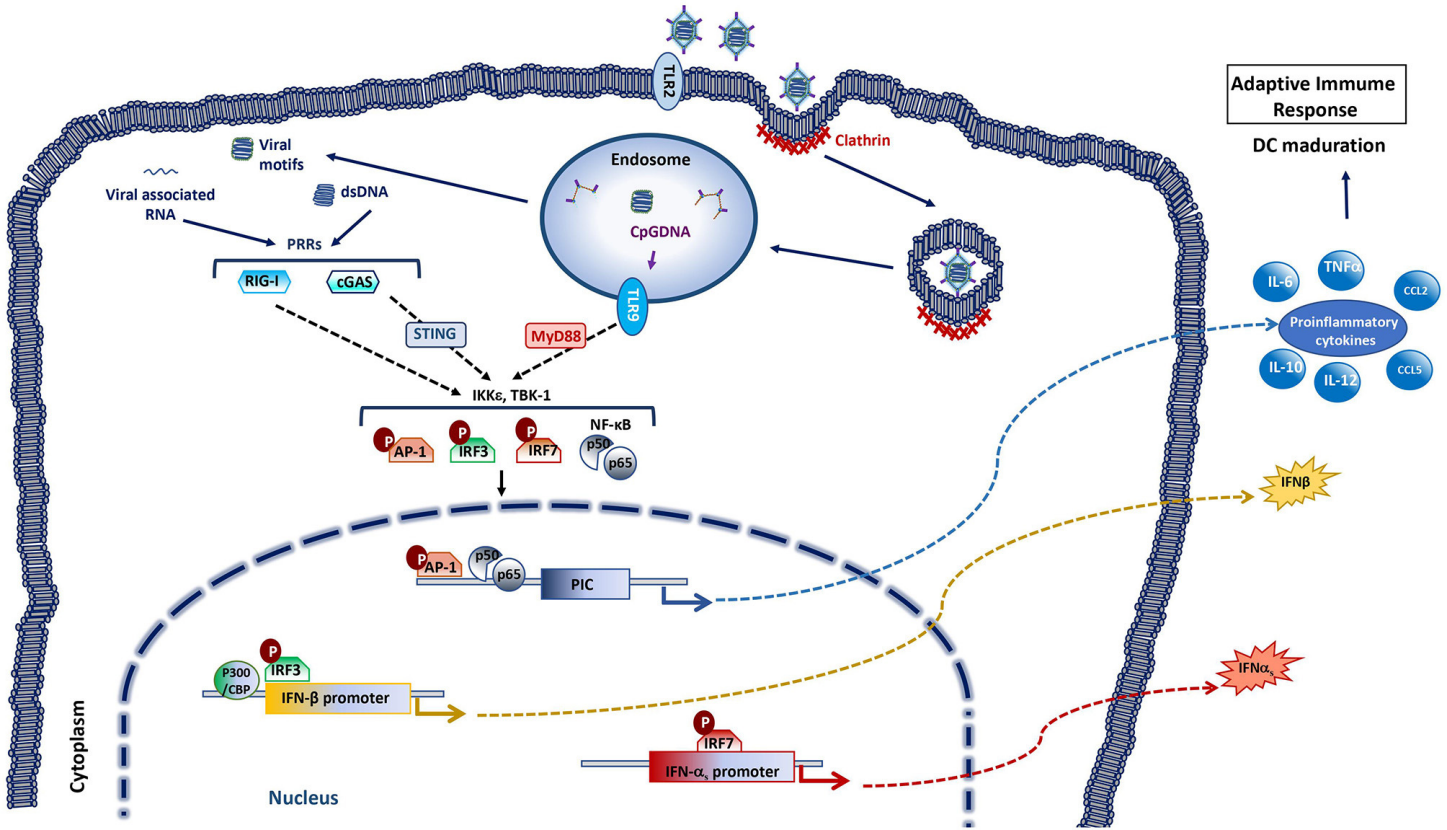
Innate immune responses activated by adenoviral vectors. The entrance of the adenovirus in the cell starts with the recognition and binding of the 12 spikes of the capsid to the specific glycoprotein receptors on the target cell membrane. This leads the invagination of cell membrane forming a pit coated by clathrin. The endocytosis process concludes with a vesicle in the cytoplasm that contains the virus inside, which will be sent to the endosome. The outer capsid of the virus disassembles with the acidification of the endosome, releasing the DNA-protein core. The viral core is liberated to the cytosol when the viral shedded spikes breach the endosomal membrane. The viral core then traffics to the nuclear pore where the genetic material is released and gene expression occurs. Systemic delivery of adenoviral vectors activates innate immune responses with secretion of pro-inflammatory cytokines and type I IFNs, through recognition of viral motifs by pattern recognition receptors (PRR). Several PRRs are involved in adenovirus recognition among these are included Toll-like receptors 2 and 9 (TLR2, TLR9), cyclic GMP-AMP synthase (cGAS) and retinoic-acid inducible gene-I (RIG-I). Adenovirus CpG DNA can be detected in the endosomes by TLR9 [that signals through myeloid differentiation primary response protein 88 (MyD88)]. Adenovirus dsDNA can be detected in the cytoplasm by the DNA sensor cGAS [that activates stimulator of IFN genes (STING)], while RNA sensor RIG-I can recognize adenovirus-associated RNAs. Recognition by PRRs triggers multiple signaling cascades (through inhibitor of nuclear factor (NF)-κB kinase ε (IKKε) and TANK binding kinase 1 (TBK-1) among others) that leads to the activation of transcription factors such as activator protein 1 (AP-1), IFN regulatory factor (IRF) 3, IRF7 or NF-κB, that promote the production of type I IFNs and pro-inflammatory cytokines. These factors promote the adjuvancy effect of the viral vector. As a result of the successive expression of exogenous and adenoviral backbone genes in the target cells, the adaptive immune response to the transgene can thus be triggered.

Adenoviral vectors, like most viruses, display different pathogen associated molecular patterns (PAMPs) that are detected by cellular sensors called pathogen recognition receptors (PRRs). Typically, the activation of PRRs leads the activation of signaling cascades that induce the expression of type I IFN and pro-inflammatory cytokines (Figure 4). These first steps from the innate immune response allow the recruitment of different innate immune cells to the site of infection which in turn help to trigger a successful adaptive immune response (117–120).

### Innate Immunity to Adenovirus Vector: The Adjuvancy Effect

Besides the adaptive immunity to the transgene that is sought with recombinant viral vector vaccines, the innate host immune responses to the viral vector can enhance the immunogenicity of the insert. Indeed, innate immune recognition of the adenoviral vector itself and its own products probably provides an adjuvancy effect. Rapid physiological responses induced by systemic adenoviral vector delivery trigger the activation of innate immunity, with induction of cytokines, inflammation, transient liver toxicity and thrombocytopenia (121–123). Adenoviruses enter the cytoplasm of the cells through different receptors, such as the Coxackie adenovirus receptor (CAR), CD46, sialic acid, integrin α*νβ*5 heparin sulfate proteoglycans, etc., depending on virus species (124–128). This entry process activates different pathways from the innate response involving toll-like receptors (TLRs), lectin receptors (LRs), autophagy, IFNs signaling, inflammasome signaling through AIM2-like receptors (ALRs), nucleotide-binding oligomerization domain (NOD)-like receptors (NLRs), and RIG-I receptors (RLRs) (Figure 4).

IFNs and anti-inflammatory cytokines activated *via* TLRs-dependent and -independent pathways constitute the typical innate immune response to adenoviruses (129, 130). TLR-2 and TLR-9 have been identified as the main activation pathways responsible for adenoviral recognition *in vivo*, leading to the production of some cytokines, such as MCP-1 and RANTES or IL6 in macrophages (130–133). In TLR2 and TLR9 -deficient mouse models, inoculation of recombinant adenovirus vector results in reduced NF-kB activation, decreased neutralization Abs production against both the adenoviral vector and the transgene, and a reduction in pro-inflammatory response and IFNα levels. The immune response to the adenovirus vector was however not completely abolished in these murine models (131), suggesting that other TLR-independent pathways are also implicated in the activation of the innate immune response against adenoviruses (134–136) (Figure 4). The IL-1α, activated through the interaction between the RGD motif of adenoviral penton base protein and β3 integrins (137) during the viral entrance is one of them. IL-1α plays an important role in inflammatory adenoviral process, which diminished in mice treated with anti-ILα antibodies and in IL1R^−/−^mice (138).

Empty adenoviral particles induce poor innate immune responses, thus the viral genomic DNA plays also an important role in innate immunity induction (132). In the cytoplasm, RLRs recognize double stranded RNAs with 5'-triphosphate groups and TLRs 3,7, and 8 recognize viral DNA and RNA on the endosomal membrane (130, 139–142). Many PRRs sense double-stranded DNA such as TLR9, IFN-γ-inducible protein 16 (IFI16), DNA-dependent activator of IRFs (DAI), DEAD (Asp-Glu-Ala-Asp) box polypeptide 41 (DDX41), cyclic guanosine monophosphate-adenosine monophosphate synthase (cGAS), and DNA-dependent protein kinase (DNA-PK) or NLRs (143–147). Type I IFN induction has been shown to depend on the cGAS-STING pathways in adenovirus infection (146), thus placing this cytoplasmic DNA sensor at the center of the cellular machinery responsible for adenovirus detection (Figure 4).

The innate immune response induced by adenoviruses involves different receptors within huge network pathways. The adenovirus serotype, the DNA incorporated and the infection milieu determine the host response and the efficacy of the vaccination based on adenoviral-based vectors. Generally, in the case of using these vectors as antigen delivery vehicle, the innate immune response induced by the adenovirus backbone, that in other applications such as gene therapies could be disadvantageous, becomes a vaccine adjuvant that helps activate an efficient transgene-specific adaptive immune response.

### Adaptive Immunity to Adenovirus Vector: A Limitation to Their Efficacy?

The adenoviral vector induces humoral and adaptive cellular immune responses. Humoral responses are mediated by neutralizing antibodies (nAb) directed against different epitopes in the hexon, fiber and penton adenoviral capsid proteins (148). The nAbs are mainly serotype-specific with no or minimal cross-neutralization capacity to other adenoviral serotypes. The serotype-specificity is mainly due to the high variability of epitopes in the hyper variable region (HVR) of the hexon protein and fiber knob among serotypes. In the cellular adaptive immune response against adenoviral vectors in humans, CD4+Th1 and CD8+ T cells against several structural adenoviral proteins have been detected. Dendritic cell (DC) infection also appears to play an important role in mounting adaptive immunity to the transgene (149). Indeed, DC infection and subsequent antigen presentation in lymph nodes is critical to establish CD8+ T cells responses.

Innate and adaptive immune response activation appears to be dose-dependent. The levels, amounts and duration of the transgene expression determine the immune responses. High transgene expression correlates with high antigen-specific cellular response (150). High and persistent transgene levels through human- or chimpanzee- adenoviral vectors in mice induce strong T cell responses but low innate immunity activation. By contrast, less potent T cell response is induced with a low transgene expression that induces a high innate immunity (150).

The magnitude of the immune response triggered by the adenoviral vector is important for the success of vaccination. The ideal situation is to induce an adjuvant response that is sufficient to mount an adaptive response against the transgene. Thus, a high transgene expression as well as high immunogenicity of the transgene product is also required to bias the adaptive immune response toward the antigen expressed by the adenoviral vector.

## Adenoviral Vector Choice to Overcome Pre-Existing Immunity

A crucial aspect in the field of recombinant adenoviral vectors is the choice of the adenovirus type. There are more than 60 serotypes in the *Adenoviridae* virus family. Many animal (sheep, cattle, swine, dogs, and monkeys) and human adenoviruses belong to the genus *Mastadenovirus*, one of the five genera (the others being *Siadenovirus, Aviadenovirus, Ichtadenovirus*, and *Atadenovirus*) included in the *Adenoviridae* family.

The adenoviral vectors most frequently used are based on human adenovirus type 5 (HAd5) and type 2 (HAd2). Their biology is well-understood and there are many commercial tools that facilitate their manipulation. One of the most important aspects to consider when choosing the virus-vector on which to base a potential vaccine is the pre-existing immunity against this virus in the host. In humans, Adenovirus serotypes 2 and 5 are the most prevalent (82%), but are nevertheless the most frequently used for vector development for clinical use. Due to the high seroprevalence of these serotypes, approaches have been developed to overcome the pre-existing immunity in humans to the backbone vector used.

The pre-existent humoral and cellular immunity against the vector reduce the expression-time of the transgene, and thereby its immunogenicity (112) due to the presence of vector-specific neutralizing antibodies in the host. After adenoviral vector administration, most of the neutralizing antibodies induced are directed against the hyper-variable loops of the viral hexon protein. Antibodies directed against conserved regions of the viral particle and capable of cross-reacting with different serotypes are also generated (151). Interestingly, existence of pre-existing immunity to the vector is not always detrimental, for instance the induction of transgene-specific CD8+T cell after a passive antibody transfer was improved in presence of pre-existing antibodies (152). Nevertheless, as pre-existing immunity remains a major throwback to the adenoviral vector development, different strategies have been developed to overcome it. The generation of chimeric adenoviral vector, replacing the HuAd5-HVR hexon sequences with the HVR from a different serotype, for instance, is one of these strategies employed to overcome pre-immunity in humans (148, 153–157). The most common strategy used to avoid seroprevalence problems is to vaccinate with a different adenoviral serotype from the seroprevalent one found in the host. Adenoviruses have broad tissue tropism but productive human infection with non-human adenoviruses is uncommon. Animal-derived adenoviruses can nonetheless infect certain human cell types and inversely, human adenoviruses are able to infect different animal organs. These cross-species infectivity characteristics of adenoviral vectors have been used for gene therapy and vaccine development. In human, different chimpanzee adenovirus have been developed as viral vectors to replace the classical HuAd5 as adenoviral vector (158–163).

In animal health, the use of human adenovirus vectors can be advantageous as animals should not have immunity to these vectors. Indeed, a human adenovirus-based vector vaccine for FMDV has been approved for cases of emergency by the FDA (164).

## Adenovirus-Based Vaccination for PPRV

Different research groups have opted for recombinant adenoviruses to generate potential vaccine vectors against PPRV by expressing F or H proteins either individually or together (33, 68, 165). Wang et al., Herbert et al., and Rojas et al. employed HuAd5 vectors deleted in the E1 and E3 regions to produce replication-defective vaccine constructs expressing the PPRV proteins (35, 37, 165). Qin et al. used a canine adenovirus only defective in the E3 region and thus capable of replication. These studies reported that vaccination induced humoral (33, 68, 165) and cellular immunity in sheep and goats against PPRV (33, 35, 37, 68).

Sheep (35) or goats (36, 37) were efficiently protected against a virulent PPRV challenge after vaccination with adenoviral-based vaccine to PPRV. Moreover, these potential vaccines overcame the known T cell immunosuppression induced by PPRV during the first days of infection (35). Analysis of immune correlates with protection has shown that immunization with adenovirus vectors expressing PPRV protein F or H can elicit cellular immunity to epitopes generated during PPRV infections (166). These vaccines also elicit neutralizing antibodies (33, 35–37, 165), although neutralizing antibody titers typically increased after virulent PPRV challenge (35–37). Thus, adenoviral vector immunization can prime the humoral and cellular response against PPRV in a manner that allows recognition of the pathogen when exposure occurs. Importantly, Herbert et al. also showed that a single immunization with HuAd5 expressing PPRV-H could protect goats from virulent PPRV challenge 15 weeks after immunization, suggesting that immunization with these vectors generates memory immune responses (37). Work to establish the duration of the memory responses induced by these vaccination strategies will need to be performed to further characterize the protective potential of these formulations.

Another aspect of immunization with recombinant adenoviruses is to choose the optimal antigen that induces protection. Wang et al. reported a slightly stronger cell-mediated immune responses and VNT titers with an adenoviral construct expressing an F-H fusion protein than with adenoviral vectors expressing either of these proteins on their own (165). Vaccination with adenoviruses expressing F or H, or a combination of both vectors appear nonetheless to produce similar levels of protection (35–37). Delivery of both immunodominant immunogen F and H is nevertheless likely to provide a broader spectrum of protection. Larger study groups will be necessary to fully evaluate the most appropriate combination of antigens that induce protective immunity. Overall, PPRV vaccinations based on adenoviral vectors have the potential to offer a DIVA vaccine solution to the field and help in disease eradication.

## Future Perspectives

The protective capacity in the natural PPRV hosts of adenovirus-based vaccines is now well-established in laboratory experiments. Adenoviruses are thermotolerant and induce potent immunity to the transgene. One of the main focuses for future research should be to establish DIVA diagnostic tests to accompany the adenovirus-based vaccines. This will help in the control and spread of the disease, but will also make these vaccines more desirable in non-endemic regions which are threatened by PPRV outbreaks. A comparative view of the experimental PPRV vaccines published in the area has been summarized in Supplementary Table 1, showing at a glance the advantages and disadvantages of these approaches. Vaccination with adenovirus-based vaccines represents a promising approach that could help combat this disease. Work nonetheless remains to be done to demonstrate efficacy in the farms and to establish the adequate protocol to provide protection to small ruminants from spreading the disease. Collaboration between the academic and the pharmaceutical sectors should be potentiated by institutions to bring to the field the advances made in PPRV vaccination based on adenoviral vectors.

## Author Contributions

JR and VM mainly wrote the manuscript. JR, NS, and VM wrote and reviewed the manuscript. All authors contributed to the article and approved the submitted version.

## Funding

This work was supported by grant RTI2018-094616-B-100 from the Spanish Ministerio de Ciencia e Innovación; grant S2018/BAA-4370-PLATESA2 from the Comunidad de Madrid (Fondo Europeo de Desarrollo Regional, FEDER) and VetBionet INFRAIA-731014 from the European Union H2020.

## Conflict of Interest

The authors declare that the research was conducted in the absence of any commercial or financial relationships that could be construed as a potential conflict of interest.

## Publisher's Note

All claims expressed in this article are solely those of the authors and do not necessarily represent those of their affiliated organizations, or those of the publisher, the editors and the reviewers. Any product that may be evaluated in this article, or claim that may be made by its manufacturer, is not guaranteed or endorsed by the publisher.
