# Supplementary material for: A New Look at Vaccine Strategies Against PPRV Focused on Adenoviral Candidates
Source: Front Vet Sci. 2021 Sep 8;8:729879. doi: 10.3389/fvets.2021.729879 (PMC8455998; doi:10.3389/fvets.2021.729879)
Supplement: Supplementary file 1 [file Table_1.DOCX]

| **Table1. Advantages and disadvantages of vaccines against PPRV** | | | | | | | | | | | | |
| --- | --- | --- | --- | --- | --- | --- | --- | --- | --- | --- | --- | --- |
| **Vaccine** | **Animal Model** | **Priming** | **Boost** | **Humoral Response** | **Cellular Response** | **Effective Protection** | **Long term checked** | **Safety** | **Adjuvant** | **Cold chain** | **DIVA** | **Refs** |
| **LA^1^** (RPV) | Goats | Negative  -NAb | - | No | ND | ✓ | 1 year | Virulent reversion risk | No | Yes | No | (166) |
| **LA^1^** (PPRV) | Goats  /Sheep | -NAb | - | Yes | Yes:  -Lympho-proliferation  -IFN-g | ✓ | 3 years | Virulent reversion risk | No | Yes | No | (56-59) |
| **LA^1^** (PPRV) | Goats | -NAb  -IgGs Anti-PPRV | - | Yes | ND | ✓ |  | Virulent reversion risk | No | No | No | (167) |
| **LA^1^** (PPRV) | Sheep | -NAb  -IgGs Anti-PPRV | - | Yes | ND | ND | ND | Virulent reversion risk | No | No | No | (167) |
|  | | | | | | | | | | | | |
| **Inactivated**  (M/08 PPRV)^2^ | Rats | No | -NAb  -IgGs Anti-N-PPRV | Yes | ND | - | ND | ✓ | Yes  ISA 71VG^3^ AFSA1  AFSA 2^4^ | No | No | (60) |
|  | Goats | -Transient  -NAb  -IgGs Anti-N-PPRV | -NAb  -IgGs Anti-N-PPRV | Yes | ND | ✓(Challenge 133 days after 1st immuniz) | ND | ✓ | Yes  (ASFA2^4^) | No | No | (60,61) |
|  | | | | | | | | | | | | |
| **DNA**  (F- or H-PPRV Semliki Forest virus replicon) | Mice | -NAb  -IgGs Anti-H-PPRV or Anti-PPRV | 2 boosters (14 days interval)  -NAb  -IgGs Anti-H-PPRV or Anti-PPRV | Yes | Yes  -Lympho-proliferation  -Cytokines detection | ND | ND | ✓ | No | No | Yes | (62,63) |
| **DNA**  (H-PPRV-Anti-idiotipic determinants plasmid) | Sheep | -NAb  -IgGs Anti-H-PPRV | -NAb  -IgGs Anti-H-PPRV | Yes | Yes  -Lympho-proliferation  -IFN-g | ND | Aprox.1 year (332 days) | ✓ | No | No | Yes | (19,64) |
|  | | | | | | | | | | | | |
| **Recombinant subunits**  **(**F- BmNPV)^5^ | Mice | - | 2 boosters  -NAb  -IgGs Anti-PPRV infected-Vero cells | Yes | ND | ND | ND | ✓ | No | Yes | Yes | (22) |
| **Recombinant subunits**  (H-PPRV Recombinant Baculovirus) | Goats | Only one immunization  -NAb  -IgGs Anti-H-PPRV | ND | Yes | Yes  -Lympho-proliferation | ND | ND | ✓ | No | No | Yes | (65) |
| **Recombinant subunits**  (H-PPRV-peanut plants) | Sheep | Oral administration (5grs)  -NAb | Five weeks at weekly intervals  -NAb | Yes | Yes  -Lympho-proliferation | ND | ND | ✓ | No | No | Yes | (23) |
|  | | | | | | | | | | | | |
| **VLPs^6,7,9^** | Mice | -IgGs^6*^  -Nab^7,8,9^  -IgGs Anti-H, F -PPRV^7,8^ and PPRV ^7,8,9^ | 2 boosters^8,9^  -Nab^6^^  -IgGs^6^^  -Nab^7,8,9^  -IgGs Anti-H, F -PPRV^7,8^ and PPRV ^7,8,9^ | Yes | ND^6^  Yes^7,8,9^:  -Lympho-proliferation^7^  -IFN-g^7,8,9^  -IL2^8^  -IL4^8,9^  -IL10^8^ | ND | ND | ✓ | Yes^6,8,9^  Freund^6,9^  AddaVax^8^  No^7^ | No | Yes | (24^6^,26^7^,27^8^,66^9^) |
| **VLPs^7,8,9^** | Goats | -Nab^7,8,9^  -IgGs Anti-H, F -PPRV and PPRV ^8,9^ | 2 boosters^8,9^  -Nab^7,8,9^  - Anti-H, F -PPRV and PPRV^8,9^ | Yes | Yes^7,8,9^  -Lympho-proliferation^7^  -IFN-g^8,9^  -IL2^8^  -IL4^8,9^  -IL10^8,9^ | ND | ND | ✓ | No^7^  AddaVax^8^  Imject^TM^  Alum^9^ | No | Yes | (26^7^, 27^8^, 66^9^) |
| **VLPs^8^** | Sheep | -NAb  -IgGs Anti-H, F -PPRV and PPRV | 2 boosters  -NAb  -IgGs Anti-H, F -PPRV and PPRV | Yes | Yes  -IFN-g  -IL2  -IL4  -IL10 | ND | ND | ✓ | AddaVax | No | Yes | (27) |
|  | | | | | | | | | | | | |
| **Reverse genetics** PPRV  recPPRV-H*^10^ | Goats | -NAb  -IgGs Anti-H-PPRV | ND | Yes | Yes | ND | ND | Virulent reversion risk | No | Yes | *In vivo* No | (30) |
| **Reverse genetics** PPRV  PPRV-VP1-FMDV^11^ | Goats | -NAb | ND | No | ND | ND | ND | Virulent reversion risk | No | Yes | Yes | (29) |
|  | | | | | | | | | | | | |
| **Vaccinia Vectors**  MVA-(F+H)-RPV | Goats | Negative  - IgGs Ant I -F, -H, -PPRV  -NAb  Low titers in half:  -IgGs Anti-PPRV | ND | No or Low | ND | ✓ | ND | ✓ | No | No | Yes | (71) |
| **Vaccinia Vectors**  MVA-F-PPRV or MVA-H -PPRV | Goats | -NAb  - IgGs Anti -H, -F | -NAb  - IgGs Anti -H, -F | Yes | ND | ✓ | 4 months | ✓ | No | No | Yes | (31) |
|  | | | | | | | | | | | | |
| **Fowl pox Vectors**  FP-F-PPRV  FP-H-PPRV | Goats | Negative  -NAb | -NAb | Low | - CD8+ IFN-g | ND | ND | ✓ | No | No | Yes | (37) |
| **Fowl pox Vectors**  FP-F-PPRV  FP-H-PPRV  + or -  Ad5-GMCSF  Ad5-IL2 | Goats | - IgGs Anti -H-PPRV | - | No | Low  - H-PPRV specific CD8+ T cells  - Lymphoproliferation Negative | ND | ND | ✓ | Ad5-GMCSF or Ad5-IL2 | No | Yes | (37) |
|  | | | | | | | | | | | | |
| **Capripox Vectors**  CPV-F-RPV or/and CPV-H-RPV | Goats | Negative  -NAb | - | No | ND | ✓ | ND | ✓ | No | No | Yes | (38) |
| **Capripox Vectors**  CPV-F-PPPV | Goats | Negative  -IgGs Anti-PPRV | - | No | ND | ✓ | ND | ✓ | No | No | Yes | (39) |
| **Capripox Vectors**  CPV-F-PPRV  CPV-H-PPRV | Goats/Sheep | -NAb  -IgGs Anti-PPRV | -NAb  -IgGs Anti-PPRV | Yes | ND | ND | ND  6 months  -NAb | ✓ | No | No | Yes | (40) |
| **Capripox Vectors**  CPV-F-PPRV +  CPV-H-PPRV | Goats | Negative:  -NAb | ND | No/ND | ND | ✓  Partial protection against PPRV in animals with previous exposure to CPV | ND | ✓ | No | No | Yes | (41) |
| **Capripox Vectors**  CPV-F-PPRV  CPV-H-PPRV  CPV-F+H-PPRV | Goats | -NAb  (CPV-F-PPRV negative) | -NAb | Yes  (higher in CPV-F+H-PPRV) | ND | ✓ | ND | ✓ | No | No | Yes | (42) |
|  | | | | | | | | | | | | |
| **BoHV-4**  BoHV-4-H-PPRV | Mice | ND | -NAb | Yes | Yes  -IFN-g  -CTL  Against H-PPRV and PPRV | ND | ND | ✓ | No | No | Yes | (43) |
| **BoHV-4**  BoHV-4-H-PPRV | Sheep | -NAb  -IgGs Anti-N-PPRV and Anti-PPRV | -NAb  -IgGs Anti-N-PPRV and Anti-PPRV | Yes | Yes  -IFN-g  Against H-PPRV and PPRV | ✓ | ND | ✓ | No | No | Yes | Personal Data submitted to publication |
|  | | | | | | | | | | | | |
| **NDV** | Goats | Negative  -NAb | -NAb | Yes | ND | ✓  (with complete vaccination regime)  Clinical signs but recuperation (one immunization) | ND | ✓ | No | No | Yes | (44) |
|  | | | | | | | | | | | | |
| **Canine Adenovirus Vector**  CAV-2-H-PPRV | Goats | -NAb  -HI^12^ | -NAb  -HI^12^ | Yes | Yes  - Lympho-proliferation | ND | IgGs detected for at least 7 months later | ✓ | No | No | Yes | (33) |
|  | | | | | | | | | | | | |
| **5-Human Adenovirus**  Ad5-H-PPRV  Ad5-F-PPRV | Mice | -NAb  - IgGs Anti-PPRV | 2 boosters:  -NAb  - IgGs Anti-PPRV | Yes | Yes:  - IFN-g (F, H, iPPRV) | ND | ND | ✓ | No | No | Yes | (68) |
| **5-Human Adenovirus**  Ad5-H-PPRV  Ad5-F-PPRV | Sheep | -NAb  - IgGs Anti-PPRV | -NAb  - IgGs Anti-PPRV | Yes | Yes  - IFN-g (F, H, iPPRV) | ✓ | ND | ✓ | No | No | Yes | (35) |
| **5-Human Adenovirus**  Ad5-F-PPRV  Ad5-H-PPRV | Goats | -NAb | -NAb | Yes | Yes  - CD8+ IFN-g | ND | ND | ✓ | No | No | Yes | (37) |
| **5-Human Adenovirus**  Ad5-F-PPRV  Ad5-H-PPRV  + or -  Ad5-GMCSF  Ad5-IL2 | Goats | -NAb (higher with GMCSF and IL-2)  - IgGs Anti -H-PPRV (higher with GMCSF and IL-2) | - | Yes | Yes  - H-PPRV specific CD8+ T cells  - Lymphoproliferation | ND | ND | ✓ | Ad5-GMCSF or Ad5-IL2 | No | Yes | (37) |
| **5-Human Adenovirus**  Ad5-H-PPRV  + or -  Ad5-GMCSF or Ad5-IL2 | Goats | -NAb  - IgGs Anti -H-PPRV | -NAb  -IgGs Anti -H-PPRV | Yes | Yes  - H-PPRV specific CD8+ T cells  - Lympho-proliferation  - CD8+ IFN-g  (higher with adj) | ✓ | 3,5 months | ✓ | Ad5-GMCSF or Ad5-IL2 | No | Yes | (37) |
| **5-Human Adenovirus**  Ad5-H-PPRV  Ad5-H-PPRV+Ad5-F-PPRV | Goats | -NAb  - IgGs Anti -H-PPRV | - | Yes | ND | ✓ | 3 months | ✓ | No | No | Yes | (36) |
| **5-Human Adenovirus**  Ad5-F-PPRV  Ad5-H-PPRV  Ad5-(F+H)-PPRV | Goats | -NAb | -NAb | Yes  (higher in Ad5-F+H) | Yes  - Lymphoproliferation  (higher in Ad5-F+H) | ND | 5 months  (NAb endure) | ✓ | No | No | Yes | (164) |

^1^ Live Attenuated

^2^ Inactivated PPRV from the Morrocco strain isolated from 2008 outbreak

^3^ Seppic, Srl, Milan, Italy

^4^ Vaxine Pty Ltd, Bedford Park, Australia

^5^ Recombinant *Bombyx mori* nucleopolyhedroviruses

^6^ Virus-like-particles formed from M, H and N PPRV proteins expressed from a baculovirus system (24). One group was inoculated with Complete Freund’s adyuvant and boosted with Incomplete Freund’s adyuvant (Sigma, St. Lours, USA) (v/v: 1/1). Another group was inoculated with the VLP without adyuvant (24).

^6*^ Specific PPRV IgGs only in the mice group immunized with adyuvant (24)

^6^^ Specific PPRV IgGs or neutralizing antibodies in both mice groups, immunized with or without adyuvant (24)

^7^ Virus-like-particles formed from M and H or M and F vaccine Nigeria75/1 PPRV strain proteins expressed through a baculovirus system (26). Without adjuvant in the immunizations (mice and goats)

^8^ Virus-like-particles formed from M and H or M and F Tibet/30 virulent PPRV strain proteins expressed through a baculovirus system (27). Immunization was performed with AddaVax adjuvant

^9^ Virus-like-particles formed from M, F, H and N PPRV (China/Tibet/geg/07-30) proteins expressed through a baculovirus system (65). In mice, immunization was performed with Complete Freund’s adyuvant and boosted with Incomplete Freund’s adyuvant (Thermo Fisher, USA). In goats, the adjuvant used in immunization and booster was Imject^TM^ Alum (Thermo Fisher, USA)

^10^ A recombinant PPRV Nigeria 75/1 vaccine strain virus with mutations in the haemagglutinin (H) gene recovered by reverse genetic (30)

^11^ A recombinant PPRV virus expressing the VP1 from FMDV recovered by reverse genetic (29): Dual lived vectored vaccine

^12^ Hemagglutination Inhibition antibody titer
